# Terahertz Spectral Properties of PEO-Based Anti-Adhesion Films Cross-Linked by Electron Beam Irradiation

**DOI:** 10.3390/polym14102008

**Published:** 2022-05-13

**Authors:** Hyeon Sang Bark, Inhee Maeng, Jin Un Kim, Kyoung Dong Kim, Jae Hun Na, Junki Min, Jungsup Byun, Yongkeun Song, Byung-youl Cha, Seung Jae Oh, Young Bin Ji

**Affiliations:** 1 Radiation Center for Ultrafast Science, Korea Atomic Energy Research Institute (KAERI), Deajeon 34057, Korea; hsbark@kaeri.re.kr; 2 YUHS-KRIBB Medical Convergence Research Institute, Yonsei University College of Medicine, Seoul 03722, Korea; inheem@yuhs.ac; 3 HW Tech, Yangsan 50585, Korea; kju710@hanmail.net (J.U.K.); chemica@nate.com (K.D.K.); 4 Gimhae Biomedical Center, Gimhae Biomedical Industry Promotion Agency (GBIA), Gimhae 50969, Korea; njhbear@gbia.or.kr (J.H.N.); mjk6412@gbia.or.kr (J.M.); jsbyun@gbia.or.kr (J.B.); songyk0314@gbia.or.kr (Y.S.); mighty20@hanmail.net (B.-y.C.)

**Keywords:** terahertz, anti-adhesion film, cross-linked, electron beam irradiation, spectroscopy

## Abstract

We investigated the spectral property changes in anti-adhesion films, which were cross-linked and surface-modified through electron beam irradiation, using terahertz time-domain spectroscopy (THz-TDS). Polyethylene oxide (PEO), which is a biocompatible and biodegradable polymer, was the main component of these anti-adhesion films being manufactured for testing. The terahertz characteristics of the films were affected by the porosity generated during the freeze-drying and compression processes of sample preparation, and this was confirmed using optical coherence tomography (OCT) imaging. An anti-adhesion polymer film made without porosity was measured by using the THz-TDS method, and it was confirmed that the refractive index and absorption coefficient were dependent on the crosslinking state. To our knowledge, this is the first experiment on the feasibility of monitoring cross-linking states using terahertz waves. The THz-TDS method has potential as a useful nondestructive technique for polymer inspection and analysis.

## 1. Introduction

The anti-adhesion barrier is a medical material used to prevent the excessive formation of fibrous tissue or their adhesion to each other in the healing process of damaged tissues caused by inflammation, wounds, surgery, etc. [1,2]. The most important properties of the anti-adhesion barrier are biocompatibility, biodegradability, and mechanical properties; therefore, film-type anti-adhesion barriers that can maximize these properties have been developed and utilized [3,4]. As a type of anti-adhesion film, anti-adhesion hydrogel films produced by dissolving various biocompatible polymers in a solvent, such as polylactic acid (PLA), polyethylene glycol (PEG), and hyaluronic acid (HA), are being actively developed [5,6,7]. Recently, synthetic polymer hydrogels have emerged as promising biomaterials for anti-adhesion films because of their biocompatibility, water absorption capability, biodegradability and better mechanical properties than natural polymer hydrogels [8]. In addition, the cross-linking method has been fundamentally applied to develop anti-adhesion films because it can improve the mechanical properties of anti-adhesion films to prevent their quick dissolution in the body [9]. The crosslinking process can additionally modify properties specific to polymer-based hydrogel films, such as the degree of swelling, mechanical strength, vapor permeability and biodegradability.

Meanwhile, polyethylene oxide (PEO) is a good polymer candidate material for anti-adhesion films because it has various advantages, such as biocompatibility, low toxicity, and water solubility. However, the use of PEO has been limited with regard to anti-adhesion films, because of its disadvantages such as weak mechanical strength, adhesion, and cell adhesion. These disadvantages of PEO have been overcome through mixing with a natural polymer (sodium alginate (SLG)) and cross-linking by a chemical approach [10]. The chemical cross-linking method is a cost-efficient and simple method, but because it requires toxic substances, such as sulfur, there are various biosafety problems caused by the resulting residual substances of this method. To solve this problem, electron beam (E-beam) irradiation was recently applied as a chemical free and simple crosslinking method [11]. This cross-linking method accelerated the hydrogel formation rates of anti-adhesion films and increased the physical strength of PEO so that the biodegradation periods of the films could be easily extended. The physical properties of anti-adhesion films can be adjusted by controlling the amount of cross-linking through the control of the E-Beam dose. Currently, the cross-linking state and its subsequent effects, such as changes in biodegradability, can be evaluated by measuring swelling through the immersion method and conducting preclinical experiments using mice [12]. These methods are invasive and nonquantitative; therefore, there is a need for a nondestructive, noncontact performance inspection method that can nondestructively monitor the degree of cross-linking of anti-adhesion films. To develop an effective anti-adhesion film, a method for monitoring the cross-linking of the anti-adhesion film is required along with cross-linking control technology.

Many studies have been reported in which anti-adhesion films are monitored by E-beam irradiation using noncontact methods, such as Fourier transform infrared spectroscopy (FT-IR), X-ray crystallography (XRD), and transmission electron microscopy (TEM), but the use of these techniques to monitor the state of cross-linking has rarely been reported. These methods are limited in regard to monitoring the cross-linking state because the energy of cross-linking is lower than that of X-rays, or the porous structures generated during the freeze-drying of the polymer composites restricts visible and infrared spectroscopy due to scattering [13,14,15]. THz waves, which are in the 0.1–10 THz frequency region, have a longer wavelength than the diameters of these porous structures. THz frequency allows not only penetration of the polymer but also the collection of the spectra of materials regardless of the scatterer, for instance, the porous structure. THz waves have been employed for spectroscopic studies of various materials, such as gases, liquids, semiconductors, proteins, and 2D materials. Moreover, since the molecular binding energy of polymers lies in the THz frequency region, spectroscopy based on the THz frequency has been used to classify the density or molecular structure of polymers, for instance, high- and low-density polyethylene. The low energy of the THz wave of 0.4–40 meV enables harmless spectroscopy for polymers that have weak molecular bonding. THz-TDS based on THz pulses yields the complex optical constants of materials directly, unlike FT-IR, which requires a fitting method to obtain complex optical constants. THz-TDS can measure both amplitude signals and phase signals, unlike FR-IR, which only measures amplitude signals. Because of this, THz-TDS has recently been applied in various industrial applications, such as food inspection, security inspection, and cancer treatment and diagnosis [16,17,18,19,20].

In this paper, we report on terahertz spectral properties of PEO-based medical films cross-linked by electron beam irradiation. This is significant because it shows the potentials for novel technique to monitor of crosslinking state in polymer materials. The role of crosslinking to improve the functionalization of polymers is very important. If more sophisticated crosslinking control becomes possible through the use of a crosslinking method by electron beam irradiation and a method that can monitor crosslinking non-destructively and quantitatively by THz-TDS, we expect that it can contribute to a breakthrough in the functionalization of polymers. We investigated the possibility of using THz-TDS to monitor the cross-linking state of anti-adhesion polymer films irradiated by an E-beam. We were able to clearly distinguish between cross-linked and noncross-linked medical films, which were manufactured with a PEO-based synthetic polymer, in our conducted experiments.

## 2. Materials and Methods

### 2.1. Materials

**Figure 1:**
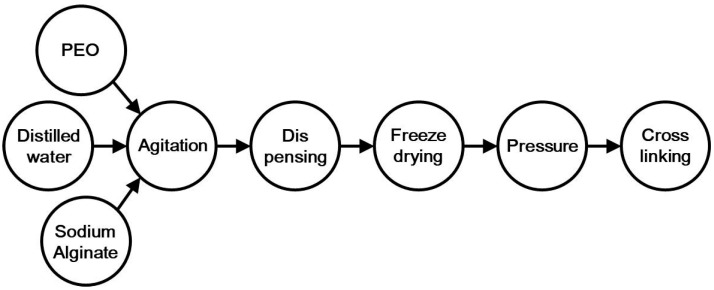
Diagram of the manufacturing process of polyethylene oxide (PEO)-based anti-adhesion films.

The manufacturing process of the polyethylene oxide (PEO)-based anti-adhesion films is divided into five steps: agitation, dispensing, freeze-drying, pressure and cross-linking, as shown in Figure 1. The first step is agitation. PEO, SLG, and sterile distilled water were mixed in a ratio of 2.1%, 0.9% and 97%, respectively. The mixed solution was stirred for 12 h to prepare an aqueous solution. In the second step, after complete agitation, 55 g of the solution was dispensed into an SUS mold using a quantitative dispenser. The third step is freeze-drying. Freeze-drying is a type of drying conducted by freezing a substance and sublimating ice directly into steam by lowering the partial pressure of water vapor. In the fourth step, the freeze-dried product was removed from the mold by pressing with an air compressor. The shape of the product was made into a film by a calendaring process using a roll-to-roll rolling machine to produce a film in the form of a sponge. The manufactured product was cut into a certain size (105 mm × 105 mm) and vacuum-packed. Finally, the film was cross-linked by irradiation with an E-beam. For E-beam processing, a commercial E-beam irradiation facility located in Daejeon, South Korea, was used. The E-beam facility generated an E-beam with an energy of 1.14 MeV and a current of 16 mA. The film was irradiated with the E-beam at various doses of 0−200 kGy by controlling the exposure time of the electron beam according to the moving speed of the conveyor belt.

**Figure 2:**
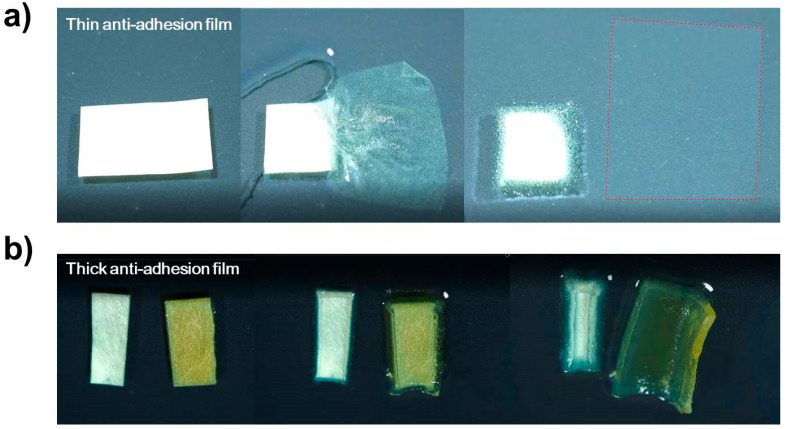
Sequence photos of the film expansion process during 25 min (thin anti-adhesion film) and 140 min (thick anti-adhesion film) after the immersion method. In the photo, the cross-linked film is on the left, and the noncross-linked film is on the right. (**a**) Thin anti-adhesion film (165 μm). (**b**) Thick anti-adhesion film (866 μm).

Figure 2 compares the expansion of the anti-adhesion film using the traditional immersion method to confirm the performance of the prepared anti-adhesion films. In Figure 2a, the thin film shows a large expansion in the horizontal and vertical directions. In Figure 2b, even in the thick film, the expansions of the cross-linked film and the noncross-linked film are clearly distinguished. It can be seen that the noncross-linked film on the left side in the photo in Figure 2 dissolves in water over time. To measure the changes in the refractive index and the absorption coefficient by monitoring the cross-linking observed in the THz region of the anti-adhesion film by using THz-TDS, the anti-adhesion film was manufactured in the same way as the end product.

### 2.2. Method (Terahertz Time-Domain Spectroscopy System)

**Figure 3:**
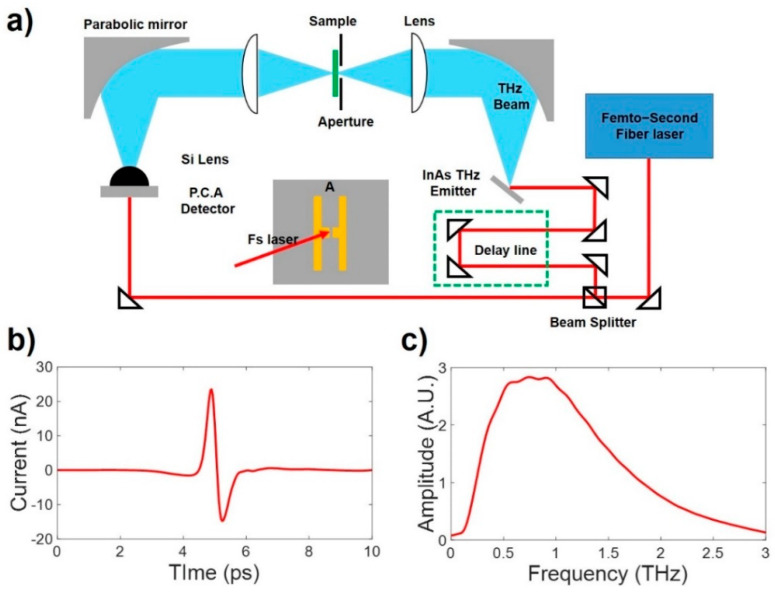
(**a**) Illustration of the THz-TDS measurement scheme. The THz pulse is generated by the InAs wafer. The THz pulse is detected by using PCA. Reference THz pulse propagation through air is measured. (**b**) Time domain signal. (**c**) Fast Fourier Transform (FFT) of (**b**).

THz-TDS was used to measure the changes in the PEO-based polymer films by E-beam irradiation. A femtosecond laser with a pulse width of 80 fs and a center frequency of 800 nm was used for the THz-TDS system [21]. The laser beam was split into two beams through a beam splitter (BS) and used for the generation and detection of THz waves. THz waves were generated by focusing a 340 mW laser pulse on a *p*-Type InAs wafer at an incident angle of 70 degrees. THz waves are generated by the photo-Dember (PD) [22] effect, in which an electric field is generated inside the InAs and the movement of carriers is caused by the surface electric field existing on the InAs surface [23]. As shown in Figure 3a, the THz waves were collimated using two parabolic mirrors. The collimated THz waves were focused by a diameter of 3 mm or less using a Plano-convex TPX lens [24]. The fs laser beam split on the BS was irradiated to a photoconductive antenna (PCA) with a 10 mW power for THz wave detection [25]. The THz waves were measured by amplifying the PCA current with a pump-probe method using a split fs laser [26]. The antenna fabricated on LT-GaAs has a line width and line spacing of 10 μm and 5 μm, respectively [27]. The dipole spacing of the antenna was 5 μm, which was used to measure broadband THz waves [28].

## 3. Results and Discussion

The anti-adhesion film was manufactured by applying a 0–200 kGy E-Beam in a vacuum-packed environment, and THz-TDS measurements were performed immediately after opening. For the THz-TDS measurements, a moisture-free environment was maintained by using dry air to remove water absorption in the THz regime [29,30]. The refractive index and absorption coefficient of each sample were calculated by using Equations (1)−(3). The reference passes through only air, and the THz signal passes through the sample. The complex frequency spectra of reference $S_{r}\left(f\right)\;$ and sample $S_{s}\left(f\right)$ are calculated using numerical Fourier transforms in the experiment. [31].

(1) $$\frac{S_{s}\left(f\right)}{S_{r}\left(f\right)}=p\left(f\right)\;e^{-j\phi \left(f\right)}$$

(2) $$n\left(f\right)=1+\frac{\phi \left(f\right)\times c_{0}}{2\pi fL}$$

(3) $$k\left(f\right)=\frac{c_{0}}{2\pi fL}\cdot \ln\left(\frac{4n\left(f\right)}{p\left(f\right)\;\cdot \;{\left(n\left(f\right)+1\right)}^{2}}\right)$$

Expressions in $n\left(f\right)$ and $k\left(f\right)$ can be written as magnitude $p\left(f\right)$ and phase $\phi \left(f\right)$ of ratio of $S_{s}\left(f\right)$ and $S_{r}\left(f\right)$. In Equation (2), The refractive index is an intrinsic constant of a material and should be independent of the thickness, so it was calculated by dividing the measured thickness value of the samples. The power absorption coefficient α (cm^−1^) can be obtained from the relationship between the imaginary part of the refractive index, $k\left(f\right)$ in Equation (3) and $k_{s}\left(f\right)=c_{0}\alpha \left(f\right)/4\pi f$ where $n\left(f\right)$ and $\alpha \left(f\right)$ denote the refractive index and absorption coefficient, respectively, and $c_{0}$, f, and L denote the speed of light, frequency, and thickness of the film, respectively. When a sample is sufficiently thick to distinguish the THz main pulse and the Fabry–Perot (FP) echo in the time domain window, the refractive index and absorption coefficient can be extracted by removing the FP echo [32]. When the THz main pulse and multiple FP echo passing through a thin sample are overlapped, the material extraction technique is required numerically [33]. In this paper, the correct refractive index and absorption coefficient values were calculated using an algorithm that finds the minimum value between the multivariate function and the measured values [34].

### 3.1. Thin Anti-Adhesion Film

**Figure 4:**
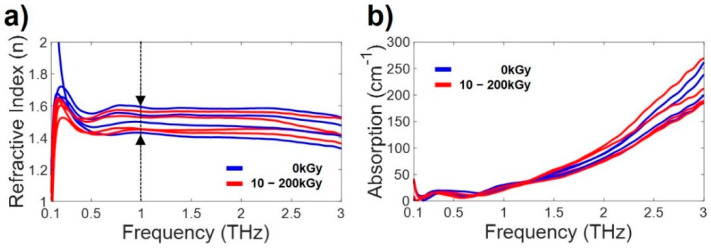
THz measurements of the thin anti-adhesion film. (**a**) Refractive indices. (**b**) Absorption coefficients. Black dot line and arrows indicate the range of refractive indices at 1 THz.

Figure 4 shows the THz-TDS measurement results of the thin anti-adhesion films. The THz-TDS measurement was repeated by changing the positions of the cross-linked and noncross-linked films. The thickness of the thin film was measured repeatedly four times and averaged, resulting in a thickness of 145–167 μm depending on the position of the film. The reference signal passing through only air and the THz signal passing through the film were measured three times and were averaged. The refractive index and absorption coefficient of the film were calculated using the average value of THz-TDS measurements. In Figure 4, the red lines (cross-linked film) and the blue lines (noncross-linked film) represent the measurement results of the same film but at different positions. Note that the difference in the refractive index and the absorption coefficient does not change, due to the thickness difference because the thickness information is included in the calculation formula and its effect on the THz measurement was removed.

**Figure 5:**
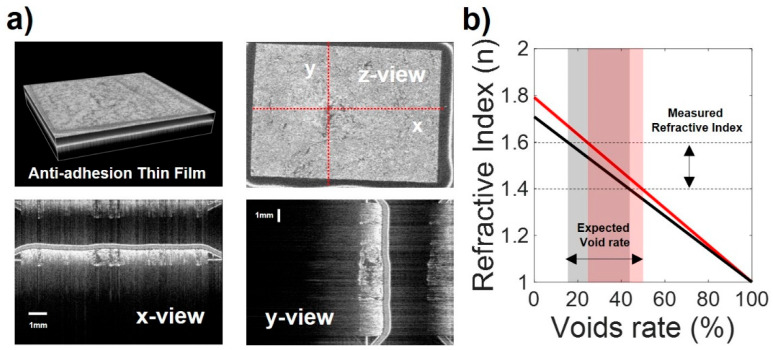
(**a**) Optical coherence tomography (OCT) images of the thin anti-adhesion film. (**b**) Graph of the measured refractive index and void ratio of the thin film. The light red region represents the void ratio of the film calculated from the refractive index of the cross-linked film (*n* = 1.791). The light black region represents the void ratio of the film from the refractive index of the noncross-linked film (*n* = 1.708).

To analyze the cause of the nonuniform refractive index and absorption coefficient of the thin anti-adhesion film, we obtained OCT images of the film, as shown in Figure 5a. 

Figure 5a shows the OCT images of the inner cross-section of the thin anti-adhesion film. The two layers in the x-y view show the cross-sections of a tape which was used to simply fix the sample on the stage and an anti-adhesion film. It can be confirmed that the empty spaces in the film are irregularly distributed compared to the tape layer. The focused THz spot size on sample surface which is measured by knife-edge measurement was 1 mm diameter. As shown in Figure 5a, the size of the pores was mm in size, so the THz measurements were inevitably affected by the pores. Because the sample manufacturing process included freeze-drying, the porous nature of the thin anti-adhesion film varied considerably, that property made the variation of the THz-TDS measurements shown in Figure 4a,b. The refractive indices of the thin anti-adhesion films were calculated between 1.4 and 1.6, as shown in Figure 4a.

**Figure 6:**
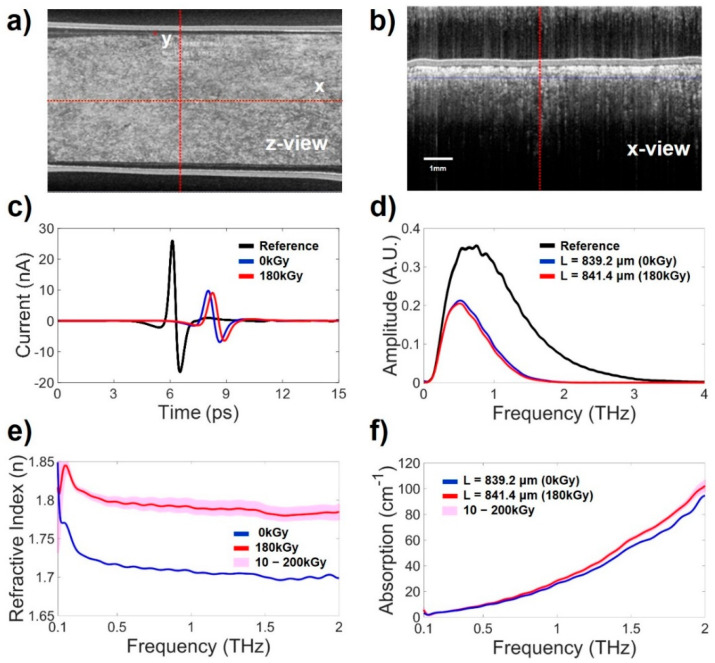
THz measurement results of the thick anti-adhesion films. (**a**,**b**) are OCT images of the thick film samples. (**c**) Time domain THz pulse signal. (**d**) THz spectrum of (**a**), which is transformed by FFT. The black line represents the THz signal passing through only air as a reference. The blue line represents the THz signal of the noncross-linked film. The red line represents the THz signal of the film cross-linked by irradiation with an E-beam at 180 kGy. (**e**) Calculated refractive indices. (**f**) Calculated absorption coefficients. The blue lines indicate the results of the noncross-linked film (0 kGy). The red lines are the THz measurements of the film cross-linked by irradiation with an E-beam 180 KGy. The pink region are the THz measurements of films cross-linked at dose of E-beam with 10–200 kGy. The variation in THz measurements according to the irradiation dose of the E-beam was small.

The void ratio of the thin film was calculated as the refractive index (1.4–1.6) of the film measured at 1 THz, indicated by the dotted line in Figure 4a. In Figure 6, the refractive indices of the cross-linked and noncross-linked thick films without voids were measured to be 1.791 and 1.708 at 1 THz, respectively. The ratio of void causing the refractive index of the thin film was calculated as the refractive index of the thick film without voids. The light red areas in Figure 5b represent cross-linked films, and the light black areas represent noncross-linked thin films. Figure 5b shows that the produced thin films do not have the same properties due to their different void ratios. Therefore, in the case of a thin film, it is difficult to measure the difference between the refractive indices and absorption coefficients of the cross-linked and the noncross-linked forms of the film.

### 3.2. Thick Anti-Adhesion Film

To overcome the problem of porosity with regard to the THz measurements, we manufactured thick-film samples. The thickness of the anti-adhesion film was controlled by adjusting the mixing ratio and pressing process; as a result, the sample was no longer porous, as shown in the inset OCT images in Figure 6. In the process of film compression, the thickness of the film increased by 700−900 μm, but a film with no voids and a uniform shape capable of resulting in accurate THz-TDS measurements was produced. The thick film samples were cross-linked by an E-beam at 0, 10, 30, 90, 120, 150, 180, and 200 kGy.

Figure 6a shows the THz-TDS signals passing through the thick films. Compared to the reference signal passing through air, the THz signals passing through the cross-linked film and the noncross-linked film clearly show a time delay and a decrease in amplitude. In the time domain, to minimize and compare the difference in time delay and signal size reduction due to the thickness of the film, the noncross-linked film (0 kGy, 839.2 μm) and the crosslinked film having the most similar thickness (180 kGy, L = 841.4 μm) were compared. The THz signal passing through the crosslinked film was measured with a 0.233 ps time delay compared to the THz signal passing through the noncross-linked film. The signal passing through the crosslinked film was more delayed even though the films were almost similar thickness, this means that the refractive index of the crosslinked film is larger than that of the noncross-linked film. This property is clearly shown in Figure 6c, in which the calculated refractive indices are depicted in the THz regime. This result shows the potential that THz-TDS can be used to distinguish the cross-linking states of anti-adhesion films that are irradiated by an E-beam. The refractive indices of the cross-linked film were approximately 0.1 greater than those of the noncross-linked film in the range of 0.1 to 2 THz.

Figure 6b shows the spectrum properties of the thick films at THz frequencies up to 4 THz. The THz signal passing through the sample exists up to approximately 2 THz due to the high absorption in the high frequency region of the films. Figure 6d shows the calculated absorption coefficient difference of the measured samples. At low frequencies (0.1–0.5 THz), there was no change due to cross-linking, but in the high frequency regime, the difference in the absorption coefficients was larger. This result also shows the potential that THz-TDS can be used to distinguish the cross-linking states of anti-adhesion films.

## 4. Conclusions

The properties of PEO-based anti-adhesion films (cross-linked and noncross-linked) were investigated nondestructively using THz waves. The refractive indices and absorption coefficients of the thin medical film showed some change when changing the measurement position. It was confirmed that the variation caused by the voids inside the sample was different depending on the position of the sample using OCT imaging. Due to the difference in the void ratio for each manufactured thin film, the measured refractive index and absorption coefficient were measured differently. Therefore, we made 700–900 μm thick medical films to minimize the effect of voids on the films. Using THz-TDS measurements, a clear difference was observed in the refractive indices and absorption coefficients of the cross-linked film and the noncross-linked film that were irradiated by an E-beam. 

To our knowledge, this is the first experiment to suggest the possibility of monitoring cross-linking states using terahertz waves. Although we have not confirmed the terahertz characteristic change according to the irradiation dose of the E-beam, this method has the potential to nondestructively monitor cross-linked and noncross-linked films. To confirm that THz-TDS is more useful as a cross-link monitoring technology, various characteristics, such as microscopic changes in electron beam irradiation dose, electron beam irradiation intensity, and moisture characteristics, will be investigated through follow-up studies. In addition, we will study how the refractive index changes according to PEO and SLG ratios in the film synthesis and will correlate these changes with mechanical properties of the film in our follow-up studies. The THz-TDS method has potential as a useful nondestructive technique for polymer inspection and analysis.

## Data Availability

The data presented in this study are available on request from the corresponding author.
